# The angiogenic response is dependent on ultrasound contrast agent concentration

**DOI:** 10.1186/2045-824X-4-10

**Published:** 2012-05-15

**Authors:** Chenara A Johnson, William D O’Brien

**Affiliations:** 1 Department of Bioengineering, University of Illinois at Urbana-Champaign, Urbana, IL 61801, USA; 2 Department of Electrical and Computer Engineering, University of Illinois at Urbana-Champaign, Urbana, IL 61801, USA; 3 Bioacoustics Research Laboratory, Department of Electrical and Computer Engineering, University of Illinois at Urbana-Champaign, 405 N. Mathews, Urbana, IL 61801, 217/333-2407, USA

**Keywords:** Angiogenesis, VEGF, Ultrasound-induced bioeffects, Ultrasound contrast agent, Proangiogenic therapy, Therapeutic ultrasound

## Abstract

**Objective:**

Ultrasound (US) and ultrasound contrast agents (UCAs) provide a way to noninvasively induce targeted angiogenesis. However, there exists a lack of understanding regarding the mechanisms of this process that has impeded progress. This study sought to characterize the angiogenic response, by both exploring the role of UCA concentration ([UCA]) in bioeffect induction at 0 days post exposure (DPE) and assessing the bioeffect as a possible potentiator of angiogenesis at 5 DPE.

**Methods:**

A 1-MHz ultrasonic transducer was used to expose the gracilis muscles of Sprague Dawley rats for 5 min with a 10-μs pulse duration, 10-Hz pulse repetition frequency, and 0.7-MPa peak rarefactional acoustic pressure (p_r_). Four [UCA]s were tested: 0x (saline), 1×, 5×, and 10×, where 1× is 5% Definity by volume of solution. Evans blue dye (EBD) was used to quantify changes in acute vascular permeability (0 DPE), and VEGF expression was quantified at 5 DPE to support that angiogenesis had occurred. CD31 staining was used to assess capillary density at both time points.

**Results:**

[UCA] was a significant parameter for determining EBD leakage (permeability) and VEGF expression (*p* < 0.001 for both). However, [UCA] was not a significant parameter for capillary density at 0 or 5 DPE. Multiple comparisons between 0 and 5 DPE showed that only 10× [UCA] at 5 DPE was significantly different than 0 DPE, suggesting a [UCA] dependence of the angiogenic response.

**Conclusions:**

This study suggests that [UCA] was a significant parameter in the induction of an angiogenic response with US and UCAs. It also suggests that rather than damage from US and UCAs, as previously speculated, a nondestructive mechanical interaction between the UCAs and vascular endothelium induces bioeffects to potentiate the angiogenic response.

## Introduction

There are presently three scenarios for which proangiogenic therapies are used clinically: chronic wounds, peripheral arterial disease and ischemic heart disease [1,2], where the treatment goal is to promote healing by inducing neovascularization. The main drawbacks for current drug, surgical, and cell-based therapies are the diffuse spread of growth factors, invasiveness, or the inability to provide spatially specific treatment. Ultrasound (US) and ultrasound contrast agents (UCAs) have been shown to provide noninvasive and spatially specific treatment, resulting in an angiogenic response to exposure [3].

Several studies report a reparative response to US and UCA exposure [4-9]. While there is a body of literature that seems to show efficacy, there is a great deal of conflicting results, perhaps because there is a lack of understanding the operative mechanisms. Recent work has shown that UCAs affect the angiogenic response by increasing expressed vascular endothelial growth factor (VEGF) [10]. In that study, however, the difference between the controls and exposed groups were significant, but subtle, possibly due to the relatively low UCA concentration ([UCA]). Current diagnostic recommendations for imaging are up to twenty times lower than concentrations used in therapeutic studies, with a wide range of [UCAs] represented in the literature [11-14].

A major impediment for progress to clinical applicability is the lack of understanding of the mechanisms that connect US and UCA to the angiogenesis response, and bioeffects to subsequent angiogenesis. [UCA] is of particular interest because increasing the [UCA] increases the number of potential cavitating bodies and the opportunity for increased bubble-bubble and bubble-vascular interactions. Many studies imply that the mechanism of US-UCA induced therapeutic effects occur via secondary wound healing; where minor, focal damage boosts normal wound healing and promotes growth of blood vessels into an ischemic area [13,15,16]. Recent work suggests otherwise, however. This study seeks to explore [UCA] in the context of bioeffect induction and subsequent angiogenic responses in an effort to establish a trail of evidence that supports that US-UCA induces bioeffects to cause angiogenesis. In addition, this study explores the role of [UCA] on those phenomena. It is hypothesized that increasing [UCA] will increase bioeffects, leading to an increased angiogenic response through a purely mechanical mechanism.

## Materials and methods

### Ultrasound exposimetry

A 1-MHz focused (f/3) single-element ultrasonic transducer (Valpey Fisher E1051, 0.75” diameter; Hopkinton, MA) connected to a power source (RAM5000, Ritec, Inc., Warwick, RI) was used for the US exposures. An established procedure for exposures was previously detailed [3]. Briefly, a custom built system containing 35°C degassed water provided coupling between the transducer and skin. An automated procedure, based on established standards [17,18], was used to routinely calibrate the US fields [19,20]. The −6-dB beamwidth and −6-dB depth of focus was measured to be 4.6 mm and 96 mm, respectively.

The *in situ* peak rarefactional pressures were estimated from p_r_ (*in situ*) = p_r_ (*in vitro*) e^-Ax^, where p_r_ (*in vitro*) is the global-maximum water-based value, and A is the attenuation of intervening layers. Prior to US exposure, the site to be exposed was measured to be equidistant (5 mm) from the inferior and superior borders of the gracilis muscle and approximately 7 mm from the distal insertion of the muscle on the tibia with an externally rotated outstretched hind limb. This site was marked and the US transducer was visually aligned with that marking. For focal depth alignment, a pulse-echo technique (low p_r_ value of 50 kPa) was used to position the focus at the surface of the muscle. After US exposure at one site, the transducer was realigned with the next US exposure site. US exposure conditions at each site were: p_r_ of 0.7 MPa (attenuation of US by 1 mm of skin is negligible [21]), pulse duration of 10 cycles (10 μs), pulse repetition frequency of 10 Hz, and exposure duration of 5 min.

### Animals

Twenty-eight female Sprague Dawley rats (Harlan, Indianapolis, IN, USA) were used to examine the role of [UCA] in the angiogenic response. Animals ranged in age from 11 to 13 weeks old and weighed between 190 and 215 g.

Rats were anesthetized with ketamine hydrochloride (87 mg/kg) and xylazine (13 mg/kg) administered intraperitoneally. Hind limb hair covering the gracilis muscles was removed with an electric clipper, followed by a depilatory agent (Nair® Carter-Wallace, Inc., New York, NY, USA) to maximize sound transmission. The rat was then placed in a custom built holder. Bilateral sites on the lateral sides of the left and right gracilis muscle were marked with a black dot to denote the US exposure location. Medial sections of the same gracilis muscle served as the control; that region was not exposed to US.

The rats were divided into two groups based on days post exposure (DPE, 0 or 5 days). For each DPE group, the rats were further divided into four [UCA]s: (0x (saline), 1×, 5×, and 10×). At 0 DPE there were four rats per [UCA]; at 5 DPE there were three rats per [UCA] (total of 28 rats). The manufacturer’s recommended dosage for infusion was used to establish the 1× concentration of Definity (Bristol-Myers Squibb Medical Imaging, North Billerica, MA, USA). The package insert states ‘1.3 mL Definity in 50 mL saline’ should be used for infusion. This [UCA] approximates to 5% Definity by volume of solution.

For 0 DPE rats, 1.5 mL of the infusion solution (containing 0, 0.07, 0.25, or 0.75 mL Definity brought to a volume with saline, for the 0×, 1×, 5×, and 10× [UCA] concentrations, respectively) was prepared in a 3-mL syringe. Evans blue dye (EBD, 10 mg/mL) was dissolved in the volume of saline prior to addition of UCA [22]. For the 5 DPE rats, the same procedure was followed without the addition of EBD due to possible dye toxicity and interference with the angiogenic progression [23].

The experimental protocol was approved by the Institutional Animal Care and Use Committee of the University of Illinois and satisfied all campus and National Institutes of Health rules for the humane use of laboratory animals. Animals were housed in an Association for Assessment and Accreditation of Laboratory Animal Care (Rockville, MD)-approved animal facility and provided food and water *ad libitum.*

### Infusion of UCAs or saline

Prior to delivery of the infusion solution by the infusion pump, the rat tail vein was manually injected for approximately 30 s with 0.5 mL of infusion solution (at 0×, 1×, 5×, or 10× [UCA]) to introduce UCAs into the circulatory system. Then, an infusion pump (model 780100; KD Scientific, Holliston, MA, USA) was used to deliver 1.0 mL of the infusion solution over 15 min into the rat tail vein at a rate of 4.0 mL/h with an approach previously used [10]. The resulting maximum infusion rates with UCAs were 3.1 × 10^7^, 1.1 × 10^8^, and 3.3 × 10^8^ microbubbles/min for 1×, 5×, and 10× [UCA], respectively.

The [UCA] was mathematically modeled to account for the UCAs half-life (estimated to be ~0.7 min in the rat), *in vivo* dissolution of Definity, duration of US exposure, accumulated UCA in the syringe, and UCAs destroyed by US exposure. The initial 30 s injection brought the [UCA] to approximately the steady-state [UCA] derived from infusion (the first and second exposed sites received equivalent [UCA]). This model assumed the recirculation rate was equal to disintegration of UCAs. The cannula was primed such that when the pump was started, UCAs entered the circulatory system. US exposure was initiated at site one approximately 5 s after the infusion pump was started (5-min US exposure per site, 3 min for realignment with the next site, and 2 sites per rat = 13 min). The 5-min each bilateral US exposures were completed while the infusion pump was still providing infusion solution. The 0x [UCA] infused rats received the same treatment but without UCA.

### Euthanization

The 0 DPE rats were euthanized within 1 h following US exposure. The 5 DPE rats were euthanized approximately 120 h after US exposure. Rats were euthanized using CO_2_ followed by cervical dislocation with prompt removal of US exposed muscle tissue via a 6-mm biopsy punch. The biopsy punch size was selected such that the entire US exposed area could be excised and the edges would not be lost in extraction. The biopsy was obtained from the same site as exposed, equidistant from the superior and inferior borders of the gracilis muscle with an externally rotated, outstretched limb.

### Tissue preparation and processing

Because the US focus’ -6-dB beamwidth was sufficiently large (4.6 mm), one-half of each exposed and control location was either preserved in RNA*later* (Quiagen, Valencia, CA, USA) (for the 5 DPE rats) or soaked in formamide for 24 h (for the 0 DPE rats). The other half was fixed for 24 h in 10% PBS formalin (Fischer Scientific, USA) for histological staining. The formalin-fixed tissues were paraffin embedded (Thermo Fisher Scientific, USA). Three-micrometer-thick sections were stained with hematoxylin and eosin (H&E) for tissue damage assessment, and with CD31 antibody (CD31, Cell Marque #1A10, Rocklin, CA, USA) for capillary density counts.

### Capillary density assessment

The biopsied muscle was stained with CD31 antibodies and used for capillary density counting using established techniques [3,10]. Only full-lumen capillaries were counted. The Carl Zeiss® Axioscope 2 upright light microscope was used for capillary density assessments (Carl Zeiss Microscopy, Thornwood, NY, USA); it had a high-power field (HPF) of 0.45 mm in diameter at 40x magnification. Fifteen HPFs were averaged and reported as capillaries/mm^2^ ± standard error of the mean (SEM). These values were normalized to the medial-site control capillary density, where a value of unity indicates equivalence to the control.

### Evans blue dye assessment for permeability

To quantify extravasated EBD, following US exposure, the skin overlaying the muscle was trimmed away and a biopsy punch was used to remove muscle from the exposed and control sites. The biopsy punch sample was placed on a paper towel for approximately 1 min to remove excess moisture and weighed; excess moisture was removed as to not bias the recorded weight of the tissue that used for EBD leakage quantification. Then half of the exposed and control sites were placed in 100% formamide for 24 h at 60°C. Formamide extraction allowed the dye to come to equilibrium with the solution. The supernatant’s absorption was measured using the Nanodrop® 2000 spectrophotometer (Thermo Scientific, Wimington, DE, USA) at 620 nm and the amount of dye determined from a calibrated standard curve [20]. The weight of the extracted tissue was recorded such that extravasated EBD could be normalized to the tissue weight (expressed as μg of EBD leaked per g of muscle). These values were then normalized to the medial-site control EBD leakage and reported as unitless values.

### Vascular endothelial growth factor (VEGF) analysis

RNA*later* preserved the RNA in the tissue for VEGF analysis. First, total RNA was isolated using the Quiagen® RNeasy kit (Quiagen, Valencia, CA, USA) via a standard protocol [24]. Then the optical density of the solution was determined using the Nanodrop® 2000 Spectrophotometer. The RNA was labeled and stored at −80°C. After isolation, the RNA was reverse transcribed to cDNA.

The real-time PCR was run on the cDNA with the ABI Prism 7500 (ABI, Applied Biosytems, Foster City, CA, USA) using a *Taq*Man One-Step RT-PCR Master Mix Reagents Kit (ABI) according to the manufacturer's recommendation. cDNA for both 18 S and vascular endothelial growth factor-A (VEGF) were used for the real-time reactions. 18 S is ubiquitously expressed across tissues and treatments, thus serves as an ideal internal control for real-time PCR. 18 S was used as a housekeeping gene whose stable expression allows relative quantification of other gene expressions. The VEGF primer was designed with the forward sequence: CCACTTCATGGGCTTTCTGCT, and reverse sequence: CACTTGTACCTCCACCATGCCAAG. VEGF expression was normalized to (quantified with respect to) 18 S in each sample and data were expressed relative to normalized values for the control in terms of fold change.

### Statistical analysis

One-way analysis of variance (ANOVA) in Matlab® (The MathWorks Inc., Natick, MA, USA) was used to determine if [UCA] were a significant parameter for EBD, VEGF expression, and capillary density. The level of significance was set at α = 0.05. Tukey-HSD multiple comparisons were used to compare different combinations of the concentrations to each other. Two-way ANOVA was used to compare the 0 DPE and 5 DPE capillary densities (excluding the control). Further, multiple comparisons between the 0 DPE and 5 DPE were performed.

## Results

### Immunohistochemistry: capillary density (CD-31)

**Figure 1:**
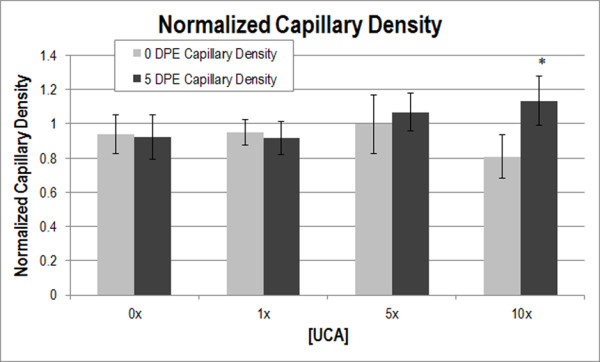
**Normalized capillary density at 0 (light grey) and 5 (dark grey) DPE.** * *p* < 0.05 with respect to 0 DPE at 10x [UCA]. The medial-site control value at 0 DPE and 5 DPE was 117.31 and 139.10 capillaries/mm^2^, respectively; these numbers were used for normalization.

Capillary density was normalized to the medial-site control value. Two-way ANOVA determined that DPE was not a significant parameter for capillary density (*p* > 0.05); however, significance was found for [UCA] (*p* < 0.05), suggesting a change in capillary density with increased [UCA] (Figure 1). The interaction term DPE X [UCA] was not significant (*p* > 0.05). Multiple comparisons between 0 and 5 DPE found the capillary density to be significantly different at 10x [UCA] (*p* < 0.05).

### Histological assessment (H&E and CD-31)

**Figure 2:**
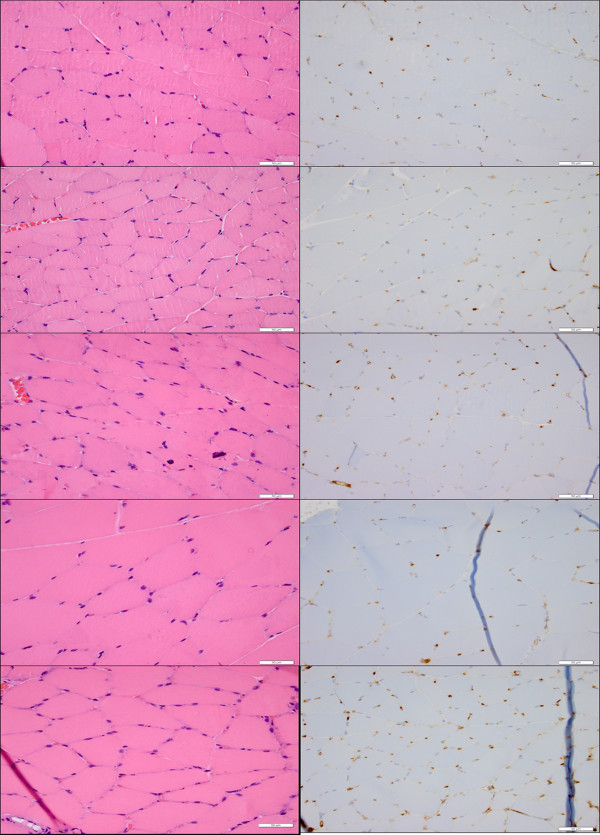
**Acute histology (H&E—left, CD31—right) demonstrating the lack of tissue level effects associated with US-UCA exposure for various [UCA] at 40× magnification.** Top to bottom: medial-site control, saline (0x [UCA]), 1× [UCA], 5× [UCA], 10× [UCA]. Bar = 50 μm. Arrows show capillary engorgement.

Tissue-level effects for 0 DPE demonstrated signs of possible capillary engorgement at 10x [UCA]. No tissue damage, inflammatory infiltrate or necrosis was observed at any of the UCA concentrations (Figure 2).

### Evans blue dye (vascular permeability)

**Figure 3:**
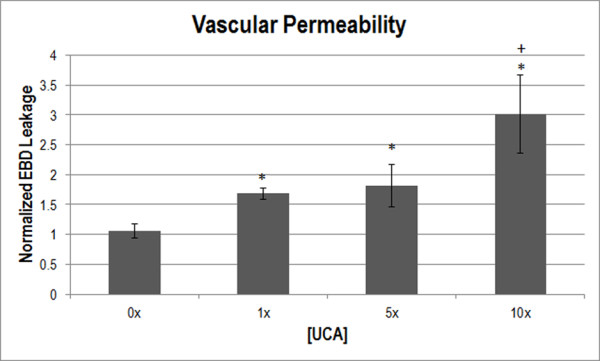
**Normalized EBD extravasation indicating vascular permeability at varying [UCA].** * *p* < 0.05 with respect to 0x [UCA], + *p* < 0.05 with respect to 1x. The medial-site control for EBD leakage was 8.84 μg of dye/g of muscle.

EBD leakage as a measurement of vascular permeability was also normalized to the medial-site control. One-way ANOVA was used to determine that [UCA] was a significant parameter for EBD leakage (*p* < 0.001) (Figure 3). The medial-site control samples were not significantly different from the saline-infused (0x [UCA]) rats (*p* > 0.05). EBD leakage demonstrated an increasing trend of permeability as the [UCA] increased, with 1x [UCA] and 5× [UCA] being near the control value for the assessment (Figure 3). Multiple comparisons between UCA concentrations determined that 1×, 5× and 10× [UCA] were significantly different from 0x [UCA] (*p* < 0.05). 10× [UCA] was significantly different from 1× and 5× [UCA] (*p* < 0.05).

### Vascular endothelial growth factor-A (VEGF)

**Figure 4:**
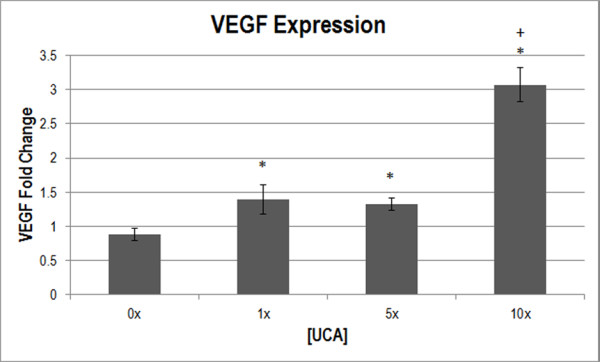
**Normalized VEGF expression (in fold change) at various [UCA]s.** * *p* < 0.05 with respect to 0x [UCA], + *p* < 0.05 with respect to 1×, 5× [UCA].

To supplement capillary density, VEGF expression was measured to assess the angiogenic response. One-way ANOVA was used to determine that [UCA] was a significant parameter for VEGF expression (*p* < 0.001). Lower [UCA]s showed less than half the VEGF expression than seen at 10x [UCA] (Figure 4). Multiple comparisons between [UCA]s indicated that 1×, 5× and 10× [UCA] were significantly different from 0x [UCA] (*p* < 0.05). 10x [UCA] was also significantly different from 1× and 5× [UCA] (*p* < 0.05).

## Discussion

The motivation for this study was to improve the understanding of US and UCA induced angiogenesis in an effort to transition the therapy to clinical use. The specific objective of this study was to determine the effect, if any, of [UCA] on the angiogenic response induced by US and UCA. Further, this study examined the acute bioeffects in an effort to provide mechanistic clues to the angiogenic response. US exposure parameters were chosen to reduce the thermal effects, with an estimated maximal temperature increase of 0.5°C [25], assuming no heat removal, which is not necessarily the case for a 5-min exposure duration. This study showed that [UCA] was a significant parameter for determining vascular permeability and VEGF expression (*p* < 0.001 for both). Multiple comparisons between 0 and 5 DPE showed that only 10x [UCA] at 5 DPE was significantly different than 0 DPE suggesting a [UCA] dependence of the angiogenic response.

Working with the hypothesis that increased [UCA] leads to increased bioeffects (without tissue-level damage) and an increased angiogenic response, the serial assessment of bioeffects and angiogenic response was carried out at several [UCA]s. In US-induced angiogenic therapy, UCA concentrations typically have exceeded the standard recommendations for imaging [4,13,26]. US and UCA therapy use [UCA]s ranging from 0 to 60% UCA by volume of solution [4,9,12-14], and the literature reports a variety of effective [UCA]s.Variability in the dosages (some [UCA]s are not reported) and differing delivery methods (*i.e.*, bolus or infusion) make it difficult to extrapolate the results to new applications [5,9,12,27].

The [UCA] range chosen herein was scaled to ensure animal survival. One study found that mouse death occurred at a concentration of 7.5 × 10^7^ microbubbles injected over 3 min [4] which equates to 3.3 × 10^9^ microbubbles/min (relative to the 3.3 × 10^8^ microbubbles/min maximum [UCA] used in this study); this information was used to determine the upper [UCA] limit. High [UCA] may also cause excess bioeffects to occur. ‘Excess bioeffects’ is defined here as a reduction in acute (0 DPE) capillary density or tissue-level changes (hemorrhage, inflammation or necrosis). One study showed that an estimated dosage of 3.2 × 10^10^ microbubbles/min (an equivalent of 16x [UCA] with the parameters used in this study) injured rat cardiomyocytes; recovery from injury was not explored [28]. Another study demonstrated an increase in capillary rupture with increasing [UCA], but after a ‘sufficiently’ high dose of UCAs was used in mice, increasing that dose did not change the response [4]. Therefore this study addressed a range of [UCA]s were explored in this study. The next step was to evaluate how [UCA] affected the acute bioeffects.

To study the bioeffects of US, EBD is frequently used. Research has shown that exposure to US and UCAs cause vessel leakage [9,25,29-31]. Microvascular effects have been shown to increase with increasing UCA dosage [27], which agrees with the findings herein (Figure 3). While UCAs were not visualized *in vivo*, the UCAs range of dynamic motions from oscillation at low p_r_ to inertial cavitation at sufficiently high p_r_ has been characterized *in vitro*[32,33]. Previous studies demonstrate that at the p_r_ used herein, the predominant UCA effect is oscillation [32,33]. Presumably, it is the hemodynamic changes induced by the UCA oscillation that perturbs the vascular endothelium leading to bioeffects. It has been speculated that US and UCAs increase vascular permeability by destabilizing the tight junctions associated with blood vessels, showing increased permeability up to 9 h after exposure [31]. Focused US, along with lipid shelled UCAs has been shown to increase EBD extravasation in muscle with as much as 8% of the total injected dose leaking at the exposed site [9]. In agreement with the literature and proposed hypothesis, increased [UCA] was associated with increased EBD extravasation (permeability bioeffect). Once permeability was assessed, the next step was to determine whether an angiogenic response occurred. VEGF and capillary density were used to assess angiogenesis.

VEGF, like US application, is known to play a role in increasing vascular permeability, therefore increases may be detected in as little as 1 h [10], which suggests that permeability could have remained elevated from the time of exposure until the angiogenic response was assessed. Shay-Salit et al*.*[34] found that VEGF receptors could be activated in approximately 2 min in bovine aortic endothelial cells exposed to shear stress or VEGF-A. US and UCAs could directly (acutely) increase VEGF or indirectly increase VEGF via shear stress from UCA oscillations that elicit permeability changes.

VEGF is necessary throughout the angiogenic process involving vascular endothelial cell migration and mitosis, and apoptosis inhibition. So, while acute increases may occur, VEGF typically peaks 3 to 7 days after insult in wound healing [35]. This study demonstrated increased VEGF at 5 DPE that correlated well with the increases in vascular permeability, displaying similar overall trends. To support VEGF expression and permeability assessments, capillary density was assessed at 0 and 5 DPE.

The 0 DPE capillary density did not significantly change with [UCA] (Figure 1), nor were there any biologically significant signs of hemorrhage, inflammation, or necrosis after exposure (Figure 2). These observations suggest that the increased vascular permeability was the result of US and UCA mechanical interactions with the vasculature causing increased porosity or increased size of pores between endothelial cells. While locally circulating cells may be damaged, the vessel lumen remains intact as demonstrated by capillary density assessments at 0 DPE (Figure 1).

Capillary density assessed at 5 DPE showed a significant difference with respect to 0 DPE for the 10x [UCA] (Figure 1), supporting the notion that sufficient increases in permeability could motivate an angiogenic response. There was no significant difference with 0x [UCA], 1× [UCA], or 5× [UCA] with multiple comparisons. This lack of significant difference (between 0 and 5 DPE) may be because the disturbance caused with lower concentrations was sufficient to statistically increase extravasation and VEGF, but not enough to biologically motivate an angiogenic response. Alternatively, the responses occurred before or after the 5 DPE time point assessment. Previous studies assessed 3 and 6 DPE time points and do not support the latter statement, however [10].

There appears to be a threshold dependence on [UCA] for the production of bioeffects, with permeability increasing from baseline at 1x [UCA]. VEGF expression was detected at 1x [UCA] as well, but the threshold was higher for the angiogenic response to be detected with capillary density (at 10x [UCA]). These findings suggest that, again while lower UCA concentrations may motivate a statistically significant response, it may be insufficient to cause angiogenesis.

## Conclusions

[UCA] demonstrates a significant effect not only in the acute bioeffects, but also in the subsequent angiogenic response. The response for both acute bioeffects and angiogenic response are positively correlated with the infused [UCA]. Beginning with a suggested mechanically induced increase in permeability, an increase in capillary density and a 3 fold change in VEGF was found. These experiments support the view that US and UCA exposure causes a mechanical effect, without tissue damage, that leads to increased microvascular permeability which induces vascular remodeling resulting in angiogenesis.

## Competing interests

The authors declare that they have no competing interests.

## Authors’ contributions

CAJ conducted the research and wrote the initial draft of the manuscript as part of her PhD thesis research. WDO guided the research and critiqued each draft of the manuscript. All authors read and approved the final manuscript.
